# “I consulted so many doctors”: the journey of tuberculosis patients in Bengaluru, India, from first symptoms to diagnosis

**DOI:** 10.1186/s12913-025-12547-6

**Published:** 2025-03-18

**Authors:** Anand D Meundi, Jan Hendrik Richardus

**Affiliations:** 1 Department of Community Medicine, Dr Chandramma Dayananda Sagar Institute of Medical Education and Research, Dayananda Sagar University, Harohalli, Kanakapura Road, Ramanagara District, Bengaluru, 562112 India; 2 Department of Public Health, Erasmus MC, University Medical Center, Rotterdam, The Netherlands

**Keywords:** Journey, TB patients, Urban, Health belief model, India

## Abstract

**Background:**

The circumstances and factors that explain diagnostic and treatment delays in TB are complex. The present study was planned to understand the journey of new pulmonary TB patients from the time they had their first symptom(s) up to the time they started treatment at a government or private health facility in Bengaluru, a metropolitan city in India.

**Methods:**

In depth interviews were conducted with twenty-six bacteriologically positive TB patients (15 male, 11 females aged 18–56 years) put on first line anti-TB treatment at government and private health facilities in Bengaluru city. Thematic content analysis of the transcript was done using the Framework approach. Constructs of the Health Belief Model were used to create codes in the framework.

**Results:**

Delays were seen in TB diagnosis and treatment in government and private sectors. Pill burden and long duration of treatment were barriers perceived by patients. Myths and lack of knowledge about TB were documented. Patients acknowledged help provided by Non-Government Organizations. All TB patients had received Direct Benefit Transfer support from the national programme.

**Conclusions:**

Empowering private providers to diagnose TB early and enabling channels for seamless referrals to a facility where anti TB treatment is provided is suggested. Tailored counselling by grass root health workers to deal with pill burden and long duration of treatment may be considered. Dissemination of knowledge about TB at community level by making it a part of agenda during routine interactions may be useful. Supporting wider engagement with non-government organizations in TB diagnosis and follow-up during treatment is recommended.

**Supplementary Information:**

The online version contains supplementary material available at 10.1186/s12913-025-12547-6.

## Introduction

The prevalence of microbiologically confirmed pulmonary tuberculosis (TB) among those aged ≥ 15 years was 316 per 100,000 population as per TB prevalence survey carried out between 2019 and 2021. This survey also uncovered the fact that about 63% of persons with symptoms has not sought health care and among those who sought, the proportion who consulted government and private providers were equal in proportion. The prevalence: notification ratio was 2.84 for India and 4.08 for Karnataka state (wherein Bengaluru, the site of the present study is situated [1]. In many parts of the world the diagnosis of TB cases and case notification has improved considerably [2]. This improvement however, has been overshadowed by the fact that many patients do not reach the end of the TB care cascade [3]. Also, unsatisfactory quality of TB care prevents patients from obtaining adequate TB services [4]. 

In India, the paths that patients need to follow for care seeking and follow-up in both the public and private sectors are difficult to connect and 50% of patients with TB symptoms seek care in the private sector [1]. Traversing these pathways is a tiring and sometimes frustrating experience. Such challenges abound in both diagnosis and treatment of TB, which is typically prolonged. Diagnosis of TB entails multiple visits to diagnostic care facilities and to the consulting physician. If treatment is not organized close by, the treatment phase also requires repeated visits for accessing medication. While these are impediments within the health system, patients face challenges outside the system as well: TB related stigma and discrimination, difficulty in accessing transport, loss of wages, and out of pocket expenditures [5]. 

Several studies in India have explored the health seeking behaviour of patients with TB symptoms in urban areas and have identified factors influencing their decision making [6–10]. In a study carried out in urban and rural areas of south India, a greater proportion of patients had approached private facility first in the urban areas compared to rural and some had changed over from government facility to a private facility owing to dissatisfaction with government facilities [6]. Similar outcomes have been recorded in a systematic review of health seeking behaviour in India. The author of this systematic review also elicited lack of perception of symptoms as severe, lack of proximity to healthcare facility and dissatisfaction with the healthcare facility as reasons for delay in seeking care [8]. Suganthi P et al. found that in Bengaluru city slums 72% of pulmonary TB patients had approached private facility first and about 87% had visited 2 or more facilities before initiating treatment [7]. Other studies have tried to quantify diagnostic and treatment delays [11, 12], and economic and social challenges during TB diagnosis and treatment [13, 14]. The circumstances and factors that explain these delays and challenges are complex and require a deeper understanding of the patients’ journey from onset of their symptoms up to the time they are placed on anti-TB treatment. With this in mind, the present study was planned with the following objectives: (a) To understand the journey of new pulmonary TB patients from the time they had their first symptom(s) up to the time they started treatment at a government or private health facility in Bengaluru (India), (b) To investigate what went well and what could have gone better in this journey, as perceived by the TB patients. The present study is among the few studies which has used qualitative methods to explore TB patient journeys and also link the identified themes with the constructs of the health belief model. Bengaluru city has been selected as the site for the present study as urban areas offer more complexities in health seeking behaviour in India [15]. 

## Materials and methods

### Study design

Qualitative study using in-depth interviews in Bengaluru city (India) between January and July 2019. The study subjects consisted of new bacteriologically positive patients with pulmonary TB, who were put on first line anti-TB treatment at government Bruhat Bengaluru Mahanagara Palike, the Bengaluru city corporation (BBMP) health centres and private clinics/hospitals. Purposive sampling was used.

### Sampling

A list of private health facilities and BBMP health centres providing TB diagnosis and treatment was prepared for each of the four zones (north, south, east, west) of Bengaluru city. Patients who were put on first line TB treatment within the last 6 months were sampled from both private and government healthcare setups, with one patient selected from each zone (north, south, east, west) to ensure representation. A total of twenty-six patients (10 in the government and 16 in the private sector) were contacted for in-depth interviews.

### Data collection

**Fig. 1:**
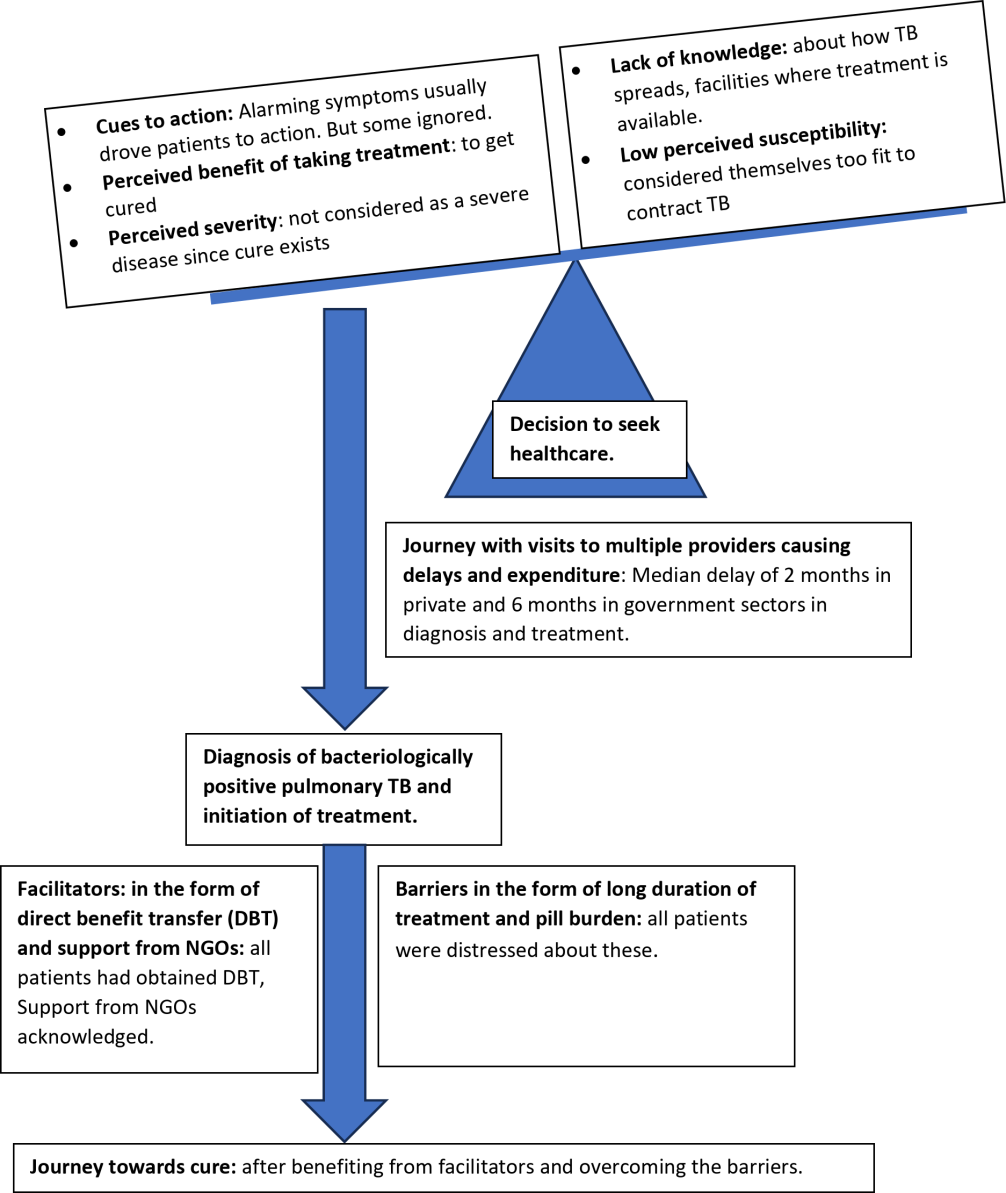
Conceptual diagram of the role of constructs of the health belief model as explanators of TB patients’ journey in this study

The patients were contacted when they visited the health centre for follow-up. In-depth interviews (IDI) were conducted with the selected patients at each of the selected health care facilities, and the process was continued ensuring that patients in all zones were contacted. The IDI interview guide is shown in Table 1 (also uploaded as supplementary file). The interview guide was pilot tested before using for interviews. The data collection was continued until thematic saturation was reached. A total of sixteen patients in the private sector and ten patients in the government sector were interviewed. The interviews were audio recorded, and transcripts written. Since the interviews were conducted in the local language Kannada, the Kannada transcripts were transliterated into English and back transliterated into Kannada to ensure consistency of content. The transliterations were done by a single linguistics expert (native Kannada speaker) with 21 years’ experience in transcription. The English version was used for analysis. Thematic content analysis was done using the Framework approach [16]. Constructs of the Health Belief Model were used to create codes in the framework [17]. The results were described based on the constructs of the health belief model (conceptual diagram, Fig. 1). Additional codes were added as the analysis progressed. The findings of the present study may be generalised to any other Indian urban area.

**Table 1:** In-depth interview guide used with TB patients put on first line anti-TB treatment at private and government health facilities in Bengaluru

Topic: The journey and obstacles faced by patients from start of symptoms up to the point they were put on treatment at a private/government facility, and what they think as facilitators that could make NTEP’s services more acceptable and accessible.
I) Main questions 1. What do you know about TB as a disease? 2. How long after you developed cough did you arrive here? 3. Can you describe all the places you visited and doctors that you consulted before being referred to this place? 4. What do you know about places where you can get treated for TB? a. What do you think about facilities for TB treatment at government and private centers/hospitals? 5. Why did you choose this place for your treatment? 6. In the whole process of diagnosis of tuberculosis up to the point when you were put on treatment here, what went well? AND what could have gone better? 7. What do you think were the changes that could have made the process of TB diagnosis a better experience? a. Describe all your expenditures related to this illness during this process. 8. Are you aware of the monthly DBT (allowance) of Rs. 500/- as nutritional support being given to patients by the government? a. If yes, what were your sources of information? b. Are you getting this benefit? c. If no, how willing are you to benefit from this allowance? d. If yes, i. in what way will it help you in completing the treatment? ii. What have your experiences been in accessing this allowance? iii. How do you think you will utilize this allowance? 9. What do you think about your regularity in taking TB treatment? a. What would be the consequences of not taking the full course of treatment? 10. What role have NGOs played in this journey of yours towards diagnosis of TB and obtaining appropriate treatment? 11. Do you have any suggestions to give to NGOs which could improve their support to patients with TB?
II) Follow-up questions as warranted
III) Probes as warranted

### Ensuring trustworthiness of data

Nvivo was used to compare codes assigned by the coders to achieve inter-coder agreement. Credibility of the data was ensured by respondent validation. Two patients who participated in the IDI were selected randomly. These selected participants were given a draft of the report of this study for their critical comments. The comments thus obtained were integrated into the analysis and interpretation. Reflexivity was applied to mitigate any effect of the presence of the first author on data collection and analysis. An audit trail was maintained to ensure confirmability. The COREQ criteria for reporting qualitative studies were adopted for presenting the results of the present study [18]. 

### Consent and ethical clearance

Written informed consent was obtained from participants of all the IDI prior to data collection. Ethical approval was obtained from institutional review board at Pariyaram Medical College, Pariyaram, Kerala where the author was working at the time of preparation of study protocol (Reference number G1.2747/12/ACME dated 20-06-2016). Data collection was undertaken between January and July 2019. Since no intervention was involved and patient confidentiality was maintained, the authors deemed it reasonable to rely on approval by the ethics committee in Kerala even though the data collection was undertaken in Karnataka.

## Results

A total of twenty-six patients were interviewed of which ten were from the government sector and sixteen from the private sector. In the government sector, the median age and age range of patients interviewed were 32 years and 20–62 years, respectively. In the private sector, the median age and age range of patients interviewed were 35.5 years and 18–56 years, respectively.

### Cue to action

Alarming symptoms such as finding blood in the sputum or vomitus and weight loss drove the patients into seeking health care. Sometimes even serious symptoms like this were neglected until they showed no signs of abatement.

I became very thin and had fever all the time. We showed to many places. (Patient 15, private hospital 48 year/male)

I had blood vomit. For 10 times, I had cough and sputum with blood. I did not take it seriously. Then I came to Bangalore, and it continued for 3 days. (Patient 5, private hospital 36 year/male)

### The journey

Multiple doctors were consulted by the patients, with a variety of treatment options offered. While the patients in the private sector took a median time of 2 months (mean 3.5 months) to reach a facility where TB was diagnosed and treatment started, those in the government sector took a median time of 6 months (mean 9.5 months) to arrive. Money spent on diagnosis and treatment from initiation of current symptoms until the patients were put on anti-TB treatment ranged from about USD (United States Dollar) 12–2400 in the private sector and USD 60–264 in the government sector. To some patients the diagnosis TB came as a shock because this was the last, they had expected. The patients in both the government and private sector had a discernible frustration regarding the number of doctors they had visited before a diagnosis of TB was made.

Many (number of doctors consulted), I cannot tell you the number. Some will give some injections…. (Patient 7, Government centre, 57 year/male)

…. I took treatment there, I had only cough remaining, all other things got cured. Then after 3 months, it again repeated. Then I became very thin. Then they took me to a private hospital and there they confirmed that I have TB. Then for one week, I took tablets. Then I had vomit. Then I started taking treatment in government hospitals. I started getting more pain. On asking there, they said pain will be there. Then from there I went to another private hospital, and they sent me here. (Patient 16, private, 26 year/male)

…. They did not even ask to do an x-ray or scan. They just kept giving tablets and I kept eating it... (Patient 15, Private, 48 year/male)

Most often the patients chose to come to a government hospital as they were either referred by private providers or because they did not have the resources to go to a private hospital. In contrast, patients landed at a private health facility most frequently because peers or family members recommended it. Responses from two patients were uncommon– one which described government hospital as dependable as the doctors took their problems seriously and another wherein a private provider had referred a patient to a government facility stating India’s commitment to end TB.

…. (I came here) because this is a government hospital, and they give good services over here. They will support the people by telling what they should do and what they should not do whereas in private hospitals, they do not take it so much seriously. I was having some other problem, and they (private providers) gave some treatment, but when I came here, they gave me very good treatment over here… (Patient 10, Government, 21 year/female).

He (private provider) is the person who directed me to go to any of the nearest government hospital and (said) they will give you better treatment for this because India is working against TB. (Patient 1, Government, 40 year/male)

All the patients in the private sector expressed immense gratitude towards social workers of NGOs, which were working as a bridge between the private facility and the National TB Elimination Programme (NTEP).

…. She (from NGO) calls up and tries to find out things and tells me that I have to take and such stuff. Those things are really important and in poor people’s cases… (Patient 12, Private, 56 year/female).

… so, they (NGO) cooperate a lot with us. I ask them why you are so bothered about my health? and I asked them whether they have any benefit. And they said they work for an NGO. (Patient 8, Private, 24 year/female)

### Patients’ knowledge about TB

Only eight patients described correctly how TB spreads from person to person. In general, it was felt that there is a need to make efforts to provide information to patients who approach private hospitals about availability of anti-TB treatment at government hospitals. There were some myths about TB that became evident. A patient mentioning probable supernatural cause of TB and a talisman as its cure was noted.

… yes, from sputum, if a fly sits on it and then comes to us, then we get it. Or if we eat the food that is eaten by pulmonary disease patients, then it will spread. (Patient 14, Private, 40 year/female)

If you drink milk, this is what will happen they said. But I do not drink milk at all. I put it in tea and drink it that’s all. They told me it is because of milk. (Patient 5, Government, 27 year/female)

I thought maybe somebody has put some evil spirits on me and so I went to the temple and wore a thayatha (talisman) also. (Patient 5, Government, 27 year/female)

### Perceived barriers in completing TB treatment

There were two main barriers elicited– one was displeasure about the number of months’ treatment they were supposed to take to bring about a cure. The other was the difficulty in consuming multiple tablets on each day of the treatment.

I feel the number of months is an irritation. If it is made less, it would be good for everyone. People suffer a lot… (Patient 6, Private, 36 year/male).

…yes, they give tablets on time, but if I see those tablets I feel like vomiting, so sometimes I miss it. (Patient 2, Government, 62 year/male)

### Perceived severity and Perceived benefit of taking treatment

All patients recognised the dangers of not taking treatment regularly, including possible death. They were aware that prompt treatment would bring about a cure and considered it a responsibility on their part to take treatment in view of dependant family. TB was not perceived as severe by the patients since it was possible to recover from it through treatment.

It is not life-threatening. If you take proper treatment, we can recover from it. That is what I have understood (Patient 4, Private, 34 year/female).

### Perceived susceptibility

TB was unforeseen by the patients, and they expressed shock over their illness. The majority replayed their previous medical histories and perceived themselves too fit to be a victim of TB. One patient seemed to be concerned about stigma attached to TB in addition to risk posed to her family members.

… soon after I retired, my medical insurance went away and generally in my family, my father and all have been very healthy. Even I have been very healthy. This has come as a shock. (Patient 12, private, 56 year/female)

…I felt why (did I get TB? ) me? kind of thing. More than what society would think, I have my brother’s children at home, what if they get infected. (Patient 4, Private, 34 year/female)

### Accessing Direct Benefit Transfer (DBT) and putting it to use

Every patient knew about the direct benefit transfer (DBT, under which, INR.1000/- is deposited from NTEP into bank accounts of patients notified in public as well as government sector as an allowance to ensure good nutrition). The NGOs had played an important role in creating awareness about this facility among patients in the private sector. All the patients interviewed had obtained the monthly allowance. They did not have any problems with accessing the monthly allowance and found the process easy. Patients in the government and private sector claimed to be using the monthly allowance to purchase food items like fresh fruits, dry fruits, eggs, and groceries.

There were no differences in the experiences of patients in the government and private sector except for the differences in delay and expenditure.

## Discussion

In depth interviews with the TB patients about their journey from symptoms to treatment uncovered phenomena that could be unravelled using the constructs of the health belief model. Using the health belief model as the framework, the present study attempted to identify critical behaviour related factors which could influence health care seeking and treatment behaviour in an urban area in India. Based on this exploration the present study has provided actionable recommendations to improve TB patient journeys in urban contexts.

Patients in the private sector had taken a much shorter time to diagnosis and treatment for TB compared to the patients in the government sector (three times longer than private sector). However, they had also spent considerably more money through their journey. Representatives of NGOs had played a crucial role as a bridge between the patients in the private sector and NTEP. Several myths existed regarding transmission of TB among patients in both sectors. Benefits of anti-TB treatment were recognised, but TB was not perceived as severe since there existed a cure. Generally, patients had not perceived themselves as being susceptible to TB until it struck. Direct benefit transfer facility was universally accessed by the patients.

Several studies have found the constructs of health belief model to be helpful in explaining prevention, care seeking, and treatment adherence behaviours in TB [19–24]. The constructs that have been used in the present study are: cues to action, perceived barriers, perceived benefits, perceived severity, and perceived susceptibility.

### The journey

Patients in the private sector had spent substantially more money than their counterparts in the public sector in their journeys documented in the present study. Comparable costs incurred by patients during TB treatment and beyond have been documented in India [13]. Another study conducted across BRICS countries (Brazil, Russia, India, China and South Africa) found that economic hardships was one among several challenges faced by TB patients during their treatment [14]. The shorter average time to TB diagnosis and treatment in the private sector was also unacceptable at around 2 months. It may be assumed that the journey in the government sector involved longer chains of care seeking. A systematic review/meta-analysis involving studies from high TB burden countries [25] and an assessment of TB patient journeys in Nepal [26] have documented visits to multiple providers to be associated with diagnostic and treatment delays. Other Indian studies have not only corroborated this finding [5, 27, 28], but also have determined that up to 30% of patients with TB could experience catastrophic expenditure [28]. Studies from Nepal, Indonesia and Myanmar have found that it was either affordability [26] or peer/family member’s/community member’s opinion and support that was responsible for the choice of facility where health care was sought [29, 30]. In the present study, similar reasons were elicited for coming to a private facility but in the case of a government facility, being referred by a private provider was the reason other than to not being able to afford private treatment. Thus, it may be construed that at least some patient journeys via private providers ended at a public facility where TB diagnosis and treatment was available. This is pertinent in view of the fact that 50% of TB patients in India had approached private providers [1]. 

All the patients in the private sector in this study expressed immense gratitude to NGOs who had helped then at various points in their journey. While recognition of NGOs by patients is one side of the coin, recognition by NTEP is the other. Recognition of NGOs by NTEP serves at least two functions– (a) motivating the NGOs to continue their good work and (b) increasing the credibility of the NGOs as authentic partners of NTEP in TB care cascade. A qualitative study carried out in Southern state in India has put on record that NGOs had played a crucial role as a link between NTEP and the private providers, and the NGOs had also perceived themselves as partners in ensuring that the NTEP reached the beneficiaries. The authors of the aforementioned study concluded that these NGOs were not recognised by the national programme for their work [31]. The authors of the present study believe that NGOs’ contribution to NTEP can be substantial and have witnessed first-hand the contribution of NGOs in Bengaluru– for instance, assisting the private providers in completing NTEP documentation and notification, assisting the patients in navigating the initial few days leading up to the diagnosis, following up with patients regarding treatment compliance and troubleshooting day-to-day issues of the patients.

### Patients’ knowledge about TB

Several myths, mostly regarding causation of TB and some regarding treatment of TB, existed among TB patients interviewed both in the private and government sectors. A great variation exists in available recent evidence regarding awareness about TB in India. While Sharma et al. found that relatively less awareness about TB causation existed in a study in Uttarakhand State of India [32], studies carried out by Dumpeti et al. in rural Telangana state [33] and by Backdahl & Sharma in Madhya Pradesh State [34] found that 80% and 93%, respectively, knew that TB was an infectious disease. Dumpeti et al. also elicited that nearly 90% of rural residents were aware about availability of free TB treatment services at government centres. In the present study however, most of the patients were not aware of this facility and wanted more information to be made available wherever they approach first– government or private facility.

### Cues to action/perceived barriers in completing treatment/perceived benefits of taking treatment/perceived susceptibility

Cues to action, are events that prompt health seeking. In the present study, serious life-threatening events pushed TB patients into seeking help. The patients expressed two main barriers in completing treatment: the long duration, and the number of tablets to be swallowed. All the patients who were interviewed recognized the benefit of taking treatment in terms of achieving a cure. Most of them did not perceive TB to be a severe disease owing to the reason that a cure exists. It was common in the interviews to find lack of perceived susceptibility. The TB diagnosis had come as a shocker.

Stigma attached to TB [35–39], long duration of treatment [37, 39], no relief from symptoms after starting treatment dissuading continuation of treatment [37] and adverse effects of treatment [37, 39] have been documented as barriers to completing treatment by recent Indian studies. Stigma was felt mostly in two contexts– in seeking healthcare and in adherence to treatment. Continuing treatment was also perceived as an obstacle for marriageability among female patients on a TB treatment. Concern for loved ones and the possibility of transferring TB to them and being motivated by relief from symptoms and further aiming at cure were common perceived benefits [37, 39]. Long duration of TB treatment being a perceived barrier has been corroborated in the present study as well. However, stigma attached to TB and adverse effects of treatment were never documented as a barrier for completing treatment in the present study.

In a study involving female students of a university in Iran, cues to action and perceived susceptibility together with health literacy and self-efficacy had predicted 52% of preventive behaviours [40]. Another Iranian study that identified determinants of TB treatment adherence came up with similar results [22]. In a study aimed at analysing intention to participate in preventive pulmonary TB chest x-ray examinations in Taiwan, perceived susceptibility, seriousness and benefits were positively associated with undergoing screening, whereas perceived barriers were negatively associated [20]. Similarly, in China, Li et al. found that perceived benefits, susceptibility and severity predicted seeking TB care [19]. In a study in Gujarat, India, pill burden was recognized as a barrier and factors resulting in reduced perception of severity, benefits and susceptibility, led to loss to follow up of TB patients [21]. Interestingly, the use of health belief model constructs to improve adherence to TB treatment was explored in Indonesia and Malaysia and the outcomes were favourable [23, 24]. 

### Accessing direct benefit transfer (DBT)

The direct benefit transfer (DBT) was launched in April 2018 in India to deal with undernutrition of TB patients. A sequential explanatory mixed methods study done in Gujarat State, India, has established that the DBT was significantly associated with favourable outcomes [41]. All TB patients in the present study had accessed DBT and did not face any hurdles in accessing the facility. A study in a district of Karnataka State in the early phase of implementation of DBT showed low coverage and substantial delays in direct benefit transfers [42]. These impediments in DBT could have been due to the fact that this study was undertaken in the early phase of implementation of DBT. In contrast the favourable experiences of patients in the present study could be attributed to the fact that the present study was undertaken in an urban area, which is also the capital of Karnataka state, where the mechanism for DBT could have been well-oiled and also to the fact that the present study was undertaken much later after implementation of DBT. The critical point to be noted here is that evidence from the present study evinces confidence that hurdles of implementation of DBT seems to have been overcome.

## Conclusions

All the patients who were interviewed in the present study exhibited favourable perceptions regarding TB with reference to the constructs of health belief model except lack of perceived severity, in addition to barriers in the form of pill burden and long duration of treatment. There was considerable delay in arriving at a facility where TB was diagnosed, and treatment initiated among both-patients put on treatment in public and private sectors. Reasons uncovered were multiple health care provider consultations, non-specific diagnostic tests and treatment provided. Further investigation into other potential barriers in the journey, designing strategies for empowering providers in the private sector to diagnose and treat TB early are recommended. Persistence of myths and lack of knowledge about TB imply that efforts must be made to increase visibility of information (regarding TB and where to seek treatment for it) at public and private healthcare facilities, in mass media and increased coverage during one-on-one interactions by grass root health workers. Reinforcement of motivation by healthcare providers to overcome barriers of pill burden and long duration of treatment is warranted. This can be achieved through regular and more frequent visits to the patients by grass root level health workers like accredited social health activists (ASHA) and male/female health workers. The assistance provided by NGOs has been duly acknowledged by patients in the present study. Gains made in ensuring that DBT reaches the beneficiaries must be maintained and enhanced.

The present study’s limitations lie in the fact that the findings of this study while generalizable to other urban areas of India, may not be generalizable to rural areas. Since the number of participants in the present study is small, quantitative estimates of delay in diagnosis and treatment and expenditure borne by the participants may not be accurate. Further research into identifying effective strategies to improve awareness of TB and its complications in the community and engaging with private providers to ensure early diagnosis and treatment of TB is needed. Based on the findings of the present study which indicate conducive role of NGOs in bridging TB patients and NTEP, deliberate efforts to collaborate with them and ensure access of all TB patients to NGOs may be considered.

## Supplementary Information

Supplementary Material 1.

Supplementary Material 2.

## Data Availability

The datasets generated and/or analysed during the current study are not publicly available due to the fact that they contain participant identifying data. However, the interview guide used in this study and the code-book have been uploaded as supplementary files.

## Abbreviations

TB: Tuberculosis
PP: Private provider
NGO: Non-Government Organization
DBT: Direct Benefit Transfer
BBMP: Bruhat Bengaluru Mahanagara Palike
IDI: In-depth Interview
COREQ: Consolidated Criteria for Reporting Qualitative Research
USD: United States Dollar
NTEP: National TB Elimination Programme
BBMP: Bruhat Bengaluru Mahanagara Palike
WHO: World Health Organization
