# Supplementary material for: “I consulted so many doctors”: the journey of tuberculosis patients in Bengaluru, India, from first symptoms to diagnosis
Source: BMC Health Serv Res. 2025 Mar 18;25:397. doi: 10.1186/s12913-025-12547-6 (PMC11916316; doi:10.1186/s12913-025-12547-6)
Supplement: Supplementary file 1 — Supplementary Material 1. [file 12913_2025_12547_MOESM1_ESM.pdf]

In-depth interview guide used with TB patients put on first line anti-TB treatment at private and government health facilities in Bengaluru.

Topic: The journey and obstacles faced by patients from start of symptoms up to the point they were put on treatment at a private/government facility, and what they think as facilitators that could make NTEP's services more acceptable and accessible.

I) Main questions

1. What do you know about TB as a disease?
2. How long after you developed cough did you arrive here?
3. Can you describe all the places you visited and doctors that you consulted before being referred to this place?
4. What do you know about places where you can get treated for TB?
   a. What do you think about facilities for TB treatment at government and private centers/hospitals?
5. Why did you choose this place for your treatment?
6. In the whole process of diagnosis of tuberculosis up to the point when you were put on treatment here, what went well? AND what could have gone better?
7. What do you think were the changes that could have made the process of TB diagnosis a better experience?
   a. Describe all your expenditures related to this illness during this process.
8. Are you aware of the monthly DBT (allowance) of Rs. 500/- as nutritional support being given to patients by the government?
   a. If yes, what were your sources of information?
   b. Are you getting this benefit?
   c. If no, how willing are you to benefit from this allowance?
   d. If yes,
      i. in what way will it help you in completing the treatment?
      ii. What have your experiences been in accessing this allowance?
      iii. How do you think you will utilize this allowance?
9. What do you think about your regularity in taking TB treatment?
   a. What would be the consequences of not taking the full course of treatment?

10. What role have NGOs played in this journey of yours towards diagnosis of TB and obtaining appropriate treatment?<br>11. Do you have any suggestions to give to NGOs which could improve their support to patients with TB?
II) Follow-up questions as warranted
III) Probes as warranted
