# Supplementary material for: “I consulted so many doctors”: the journey of tuberculosis patients in Bengaluru, India, from first symptoms to diagnosis
Source: BMC Health Serv Res. 2025 Mar 18;25:397. doi: 10.1186/s12913-025-12547-6 (PMC11916316; doi:10.1186/s12913-025-12547-6)
Supplement: Supplementary file 2 — Supplementary Material 2. [file 12913_2025_12547_MOESM2_ESM.pdf]

Qualitative TB patient interviews

Codes

Codes and sub-codes
Cue to action
Factors influencing care seeking decision.
Knowledge of TB as a disease
Fear of TB
Knowledge about consequences of not completing treatment
Knowledge about where treatment for TB is given
Perceived barriers
Access to health care facility
Contextual factors
Transportation issues

Codes and sub-codes
Perceived benefit
Perceived severity
Perceived susceptibility
Perceived general powerlessness
Perceptions about GOVERNMENT facilities for TB treatment
Avoiding health care services.
Distrust in health system
Ineffective health system
Perceptions about PRIVATE facilities for TB treatment
Receiving monthly allowance
Experiences in accessing the monthly allowance
how will you use it
Knowledge about monthly allowance

Codes and sub-codes
Sources of information
willingness to receive monthly allowance
Resorting to self treatment
Self efficacy
Perceived regularity in taking anti TB treatment
The journey
Did NGOs play any role in your journey
Suggestions to NGOs to improve
expenditures incurred
Experiences of accessing health care
Reasons for coming to government centre

Codes and sub-codes
Reasons for coming to private centre
Time taken to arrive
Could have gone better
went well
